# Investigating key elements of digital resilience among nursing undergraduates: a qualitative study

**DOI:** 10.3389/fmed.2024.1452580

**Published:** 2025-01-31

**Authors:** Fanfan Li, Qiuping Ma, Chunxiao Yang, Mingyang Zhong

**Affiliations:** ^1^Faculty of Nursing, Guangxi University of Chinese Medicine, Nanning, China; ^2^Higher Vocational and Technical College, Guangxi University of Chinese Medicine, Nanning, China

**Keywords:** e-learning, nursing practice, qualitative research, key elements, digital resilience, nursing undergraduates

## Abstract

**Objective:**

To explore key elements of digital resilience in nursing undergraduates, providing a foundation for comprehensive assessment and training during medical colleges’ digital transformation.

**Methods:**

Conducted semi-structured interviews with 20 nursing undergraduates experienced in online learning or digital resource use from March–May 2024, utilizing descriptive qualitative research and directed content analysis.

**Results:**

Identified five themes with nineteen sub-themes: understanding digital threats (information overload, decreased learning engagement, impaired social interaction, digital technology failures, digital security risks), knowing coping strategies (seeking teacher support, seeking peer support, seeking social support), learning knowledge and skills (nursing expertise, autonomous learning ability, digital technology application skills, digital learning skills, digital communication skills), overcoming digital threats stress (psychological resilience, learning perseverance), and adapt to digital environment (self-regulation and motivational efficacy, online learning self-efficacy, digital skills self-efficacy, social interaction self-efficacy).

**Conclusion:**

Nursing undergraduates’ digital resilience is multifaceted. Medical colleges should strengthen these aspects to empower students to confidently navigate digital risks and adapt to educational and healthcare digital transformation.

## 1 Introduction

Since the inception of the “Digital China” strategy within the 13th Five-Year Plan framework, digital technology has seamlessly infiltrated two crucial sectors: education and healthcare, markedly propelling their digital transformation. This accelerated transformation in education has prompted the widespread embrace of online learning in nursing education, establishing it as a prevalent norm and fostering the profound application of digital resources and tools within the field (1). Consequently, this shift presents nursing undergraduates with novel and heightened demands and challenges, necessitating the enhancement of their comprehensive abilities. In parallel, the digital revolution in healthcare has catalyzed innovation in nursing models, emphasizing the need for future nurses to acquire interdisciplinary knowledge to adeptly navigate clinical care amidst the dynamic shifts in digital technology (2, 3). This underscores the imperative for nursing professionals to continuously adapt and upgrade their skills to meet the evolving requirements of the digital era in both educational and healthcare settings.

Upon engaging in online learning and utilizing digital resources and tools, nursing undergraduates confront adaptive challenges that culminate in decreased learning engagement, diminished self-regulated learning abilities, weakened psychological belonging, and escalated psychological stress, ultimately leading to issues such as online anxiety and depression (4–6). Digital resilience is the capacity and dynamic cycle process of an individual to change their behavioral performance and psychological functioning through understanding the threats, knowing approaches, learning knowledge and skills, recovering from stress, and moving forward when facing various digital technology-related threats within the school education settings (7). This resilience facilitates the precise identification and effective management of threats posed by online learning and digital technology, fostering efficient and healthy learning patterns that enhance overall well-being. Consequently, actively developing digital resilience is imperative for nursing undergraduates.

Currently, both international and domestic academic circles are dedicated to conducting a series of in-depth studies with the aim of comprehensively exploring the constituent elements of digital resilience for specific groups. Specifically, Grant and Clarke (8) have systematically identified five core elements required for electronic workers from the perspective of the capabilities needed in the digital age. These elements include social and relational skills, trust, knowledge, personal efficiency, and self-protection. These core elements have been further refined into 21 sub-elements, which not only provide clear guidance for electronic workers in managing the pressures brought about by the integration of digital technology into their daily lives and work but also offer practical pathways for organizations and management to more effectively support and develop the digital resilience of electronic workers.

In contrast, Zhong and Liu (9) have adopted a different approach. Starting from the attitudes and beliefs of teachers, they have closely focused on the core of teachers’ problem-solving abilities. By combining existing research findings on psychological resilience, they have deeply explored the constituent elements of teachers’ digital resilience. Their findings indicate that teachers’ digital resilience primarily encompasses four elements: adapting to the environment, responding to challenges, self-regulation, and seeking support. These elements are designed to assist teachers in better adapting to the new paradigm of educational practice under digital transformation. Furthermore, they aim to promote the deep integration of digital resilience with professional competence, thereby comprehensively enhancing teachers’ subject teaching and educational abilities.

Furthermore, Xue (10) has systematically examined the actual needs of junior high school students in the context of online learning. Through rigorous research, they have elucidated the defining characteristics of digital resilience among this demographic, which encompass self-regulation, positive cognition, emotional control, and the active seeking of support. These characteristics have been further refined into ten distinct sub-elements. The findings of this research provide educators with a solid scientific foundation upon which to develop effective strategies aimed at enhancing the digital resilience of junior high school students within online learning environments.

Additionally, Chen (11) has focused his attention on the high-risk group of college students within the digital realm, taking into consideration their cognitive development levels. Through in-depth analysis, he has identified the key elements of digital resilience among college students, which primarily include tool use, information acquisition, social participation, and creation and publication. These elements have been further elaborated into twenty sub-elements. The research findings offer a robust theoretical framework and practical guidance for the assessment and cultivation of digital resilience among college students, serving as a valuable resource for educators and policymakers alike.

In summary, research endeavors pertaining to digital resilience have predominantly centered on groups such as middle school students, college students, and social workers. However, it is pertinent to note that nursing undergraduates, who are frequently enmeshed in digital technology and confront substantial digital challenges and threats, have not garnered sufficient attention in the realm of digital resilience research, thereby constituting a notable void in the existing scholarly literature. A considerable variance is evident in the awareness levels of nursing undergraduates concerning their own digital resilience, which has a pronounced impact on their nurturing and professional development. Consequently, there is an urgent and pressing need to delve into the components of digital resilience among nursing undergraduates within the context of online learning and digital nursing practice. This exploration is vital for advancing and refining the theoretical framework of digital resilience, and for establishing a robust foundation for subsequent empirical investigations.

The conceptual model of digital resilience has unveiled intricate relationships among attributes, antecedents, and consequences, and has been extensively utilized to explore the multifaceted elements of digital resilience (9, 11). In order to address this research gap concerning nursing undergraduates’ digital resilience, this study adopts a descriptive qualitative methodology to conduct a preliminary and exploratory investigation into the characteristics of digital resilience among undergraduate nursing students. The ultimate objective of this endeavor is to furnish pivotal references for the training and development of nursing undergraduates in the digital transformation of education and healthcare. By doing so, it aims to empower them to more effectively navigate and overcome challenges in the digital age, ultimately facilitating their comprehensive personal and professional growth.

## 2 Methods

### 2.1 Study design

A descriptive qualitative design was utilized, which inherently enables the portrayal and comprehension of participants’ lived experiences, rooted in their individual perceptions (12). Consequently, this study employed this methodology to articulate nursing undergraduates’ insights and perspectives on the particular attributes of their personal digital resilience, essential in the context of nursing education and the digital transformation of healthcare.

### 2.2 Setting-participants

Between March and May 2024, a deliberate purposive sampling strategy was adopted to select nursing undergraduates spanning their academic journey from the first to the fourth year at a renowned medical university situated in the Guangxi Zhuang Autonomous Region, constituting the focal interview population. The inclusion criteria meticulously outlined: (1) a commitment to voluntary participation in the interviews, (2) a background in online learning or proficiency in utilizing digital resources and tools, and (3) full-time enrollment in an educational program. Conversely, the exclusion criteria rigorously enforced: (1) any withdrawal from the study during the interview phase and (2) concurrent involvement in other analogous research endeavors. Consequently, a cohort of 20 nursing undergraduates was successfully recruited and each member was numbered in the predetermined order, from N1 to N20. Table 1 provides an overview of the participants’ general information.

**Table 1:** General information of the participants (*n* = 20).

Participants	Sex	Age	Grade	Online learning experience	Experience with digital resources and tool applications	Interview method	Interview location
N1	Female	21	Junior class	Yes	Yes	Offline interview	Classroom Room 1–114
N2	Female	21	Junior class	Yes	Yes	Offline interview	Classroom Room 1–114
N3	Female	21	Junior class	Yes	Yes	Offline interview	Classroom Room 1–114
N4	Female	20	Second grade	Yes	Yes	Offline interview	Classroom Room 1–114
N5	Male	20	Second grade	Yes	Yes	Offline interview	Classroom Room 1–114
N6	Female	20	Second grade	Yes	Yes	Offline interview	Classroom Room 1–114
N7	Female	20	Second grade	Yes	Yes	Offline interview	Classroom Room 1–114
N8	Female	20	Second grade	Yes	Yes	Offline interview	Classroom Room 1–114
N9	Male	21	Second grade	Yes	Yes	Offline interview	Classroom Room 1–114
N10	Female	20	Second grade	Yes	Yes	Offline interview	Classroom Room 1–114
N11	Female	20	Second grade	Yes	Yes	Offline interview	Classroom Room 1–114
N12	Female	20	Second grade	Yes	Yes	Offline interview	Classroom Room 1–114
N13	Female	20	Second grade	Yes	Yes	Offline interview	Classroom Room 1–114
N14	Female	22	Senior class	Yes	Yes	Offline interview	Classroom Room 1–114
N15	Female	22	Senior class	Yes	Yes	Offline interview	Classroom Room 1–114
N16	Female	22	Senior class	Yes	Yes	Online interview	Tencent conference
N17	Male	22	Senior class	Yes	Yes	Online interview	Tencent conference
N18	Female	19	First grade	Yes	Yes	Online interview	Tencent conference
N19	Female	19	First grade	Yes	Yes	Online interview	Tencent conference
N20	Female	19	First grade	Yes	Yes	Online interview	Tencent conference

### 2.3 Establishment of a research team

The research team comprises a master’s degree supervisor and three nursing master’s degree students, all currently pursuing their studies. Each team member boasts experience in online learning and proficiency with digital resources and tools. The supervisor oversees resource coordination and interview outline design, whereas the students undertake literature review, interview conduct, data analysis, and research paper writing.

### 2.4 Develop the interview outline

The research team employed the five pivotal elements outlined in the “Preliminary Conceptual Model of Digital Resilience” (7), namely: understanding online threats (pertaining to individuals’ perception of potential challenges and threats within the online environment), knowing solutions (reflecting individuals’ proficiency in utilizing strategies and resources to address digital risks), learning knowledge and skills (indicating individuals actively seeking knowledge and skills to manage digital risks through personal online learning experiences), recovering from stress (concerning individuals’ self-regulation to revert to a normal learning state after encountering negative events stemming from digital risks), and advancing through self-efficacy (whereby individuals motivate themselves to effectively tackle online risks based on a strong belief in their abilities). After undergoing a rigorous literature review and expert consultation, the research team, based on this comprehensive foundation, initially formulated a semi-structured interview outline. The specific content of this outline is detailed in Table 2.

**Table 2:** Preliminary semi-structured interview outline.

Items	Questions
1	How often do you participate in online learning or use digital resources and tools?
2	What threats do you perceive when participating in online learning or using digital resources and tools?
3	Do you know how to seek help when faced with threats?
4	What efforts have you made to adjust with these threats?
5	Can you self-evaluate your adaptability in the face of these threats?
6	What psychological characteristics do you think you should possess to effectively cope with threats?

Subsequently, a master’s degree candidate in nursing conducted a series of preliminary interviews with three undergraduate nursing students. It is noteworthy that, despite the effort invested, the results obtained from these interviews were ultimately excluded from the final statistical analysis. Taking into account the feedback garnered from these preliminary interviews, as well as the insightful professional perspectives imparted by the interviewees, the research team meticulously adjusted and refined items 3, 4, and 5 of the interview outline. The primary objective of this refinement process was to enhance the relevance and sensitivity of the questions, ensuring that they were in complete alignment with the core elements of the study. As a result of these adjustments, the respondents were guided to provide clearer and more concise responses, which facilitated a more accurate and insightful understanding of the research topic. Consequently, the interview outline was finalized, with its comprehensive content encapsulated in Table 3.

**Table 3:** Final semi-structured interview outline.

Items	Questions
1	How often do you participate in online learning or use digital resources and tools?
2	What threats do you perceive when participating in online learning or using digital resources and tools?
3	What methods do you usually adopt to cope with threats?
4	What knowledge and skills do you think you should possess to effectively cope with threats?
5	What is your psychological journey when facing threats?
6	What psychological characteristics do you think you should possess to effectively cope with threats?

### 2.5 Data collection

The data was gathered via a semi-structured interview method, adhering to a one-on-one format. Prior to the interviews, the participants were apprised of the interview’s objectives, content, and methodology, and a mutually agreeable time, venue, and format were established. Each interview was conducted within a time frame of 15 to 30 min, with the exception of N16–N20, who participated via online means; the remainder took place face-to-face in a serene and secluded classroom atmosphere. Prior to the official interview commencement, participants were mandated to sign an informed consent form or verbally consent to participate, and pertinent aspects of their verbal and non-verbal interactions were diligently recorded in the interview memorandum. The determination of the interview sample size was primarily contingent upon the content discoveries made during the data analysis process (13). Specifically, saturation was deemed to have been achieved when data analysis yielded only content that replicated existing codes or themes, without revealing any novel codes or themes. Concurrently, we also evaluated data saturation based on the completeness of the identified codes or themes. The establishment of this criterion is aimed at ensuring a profound comprehension of the interview data, ultimately facilitating a more comprehensive and rigorous analytical process.

### 2.6 Data analysis

Upon the conclusion of the interview, two nursing master’s degree students, currently enrolled in their academic pursuits, promptly transcribed the interview recordings into textual materials within a 24 h time frame. Subsequently, they provided the organized content back to the interviewees for verification. These textual materials were then stored and rigorously analyzed using Nvivo14.0 software. The analysis of the interview content adhered strictly to a directed content analysis approach (14), involving the following methodical steps: (1) Utilizing preliminary literature research, essential concepts and variables were identified and employed as the initial coding categories, with operational definitions defined for each category. (2) The interview materials were thoroughly and meticulously reviewed, with particular emphasis on marking statements that were intimately tied to the research questions. (3) The initial coding scheme was applied to code and categorize the content, with new codes assigned to uncoded content and adjustments made to the existing coding scheme as necessary. (4) The codes were subsequently classified based on their relevance, leading to the formation of coherent categories and subcategories. (5) Finally, the categories were organized and conceptually framed to derive meaningful themes.

### 2.7 Quality control

After systematically studying courses on qualitative research methodologies and engaging in pertinent research projects, the researcher has attained a proficient command of the methods and interview tactics in qualitative research. Acknowledging the heterogeneity among research participants, this study selectively enrolled undergraduate nursing students across various academic levels. The data analysis was rigorously conducted through the consensus approach, with the research team collaboratively resolving any contentious issues. The final report conforms to the Consolidated Criteria for Reporting Qualitative Research (15), guaranteeing a comprehensive and transparent account that exemplifies the stringent standards of qualitative nursing research.

## 3 Results

Having achieved a notable milestone, this study has comprehensively completed an aggregate of 20 meticulous in-depth interviews with a deliberately crafted sample cohort comprising 20 nursing undergraduates. The cohort displays a distinctive gender ratio, with 3 males and 17 females, further highlighting its multifaceted nature. The grade distribution within this cohort is equally varied, spanning across the academic spectrum, encompassing 3 first-year students, 10 s-year students, 3 third-year students, and 4 fourth-year students, ensuring a comprehensive representation of the student population. The cumulative interview duration of 348 min ensures the profoundness and intricacy of the gathered data. The interview content has been diligently structured and transcribed, totaling 43,484 Chinese characters, followed by a rigorous coding process, resulting in the identification of 295 items. Upon thorough examination and analysis of these transcripts and coded items, the study adeptly distilled and synthesized the pivotal traits of digital resilience displayed by nursing undergraduates. These attributes were meticulously categorized into 5 core themes, further elaborated into 19 sub-themes, offering a comprehensive and nuanced insight into the adaptability and resilience of nursing students amidst a digital landscape.

### 3.1 Theme 1: understanding digital threats

#### 3.1.1 Information overload

During the interview process, a substantial majority of nursing undergraduates reported encountering the challenge of information overload when engaging in online learning and utilizing digital resources and tools.

N5: Online learning platforms offer diverse materials transcending professional curricula, but this variety complicates knowledge understanding and assimilation. N16: Websites abound with perplexing information, hindering the ability to condense and refine it, and posing challenges in assessing source credibility.

#### 3.1.2 Decreased learning engagement

Several nursing undergraduates have reported that their engagement in learning has been directly affected by the extended duration of online learning, as well as their unfamiliarity with digital resources and tools.

N10: Prolonged online learning has exhausted me, hindering my ability to sustain energy while studying course content. N18: Unfamiliarity with the learning platform and tools has diminished my enthusiasm and efficiency, impeding my focus on completing learning tasks.

#### 3.1.3 Digital technology failures

Nursing undergraduates emphasized, during interviews, that their most frequent issues with online learning and digital resource utilization pertained to digital technology failures, specifically network delays and operational errors, significantly impacting their learning experience.

N3: During the online learning process, issues pertaining to network lag hinder my synchronization with the teacher’s interface, consequently impeding a smooth follow-up of the teaching pace. N19: When utilizing certain learning platforms and tools, I often encounter challenges and have a keen desire for guidance from others.

#### 3.1.4 Impaired social interaction

During interviews, nursing undergraduates have reported a decrease in interaction frequency with teachers and classmates, attributed to the prevalence of online learning models and their unfamiliarity with digital resources and tools.

N6: In the realm of online learning, environmental constraints impede my capacity for real-time interaction with teachers and hinder the accessibility of prompt tutoring. N11: During online group collaborations, I discern a notable inadequacy in communication among teammates, significantly impeding the attainment of optimal collaborative outcomes.

#### 3.1.5 Digital security risks

Nursing undergraduates have reported digital security breaches involving personal info leaks during online learning & digital resource usage.

N9: Upon logging in, several learning platforms display intrusive ads, risking malware downloads from mistaken clicks, posing a security threat. N15: During browsing, I occasionally encounter high-risk sites, exposing my computer to viruses, disrupting system functions, and hindering file storage.

### 3.2 Theme 2: knowing coping strategies

#### 3.2.1 Seeking teacher support

Most nursing undergraduates view teacher support as key to tackling digital risks.

N2: When interest fades in online learning, I privately consult the teacher during breaks, seeking methods to reignite my passion. N8: Unable to find or comprehend required info, I promptly ask the teacher to share resources.

#### 3.2.2 Seeking peer support

Nursing undergraduates suggest peer support, understanding, and assistance as a strategy for digital risk management.

N6: Whenever I encounter an unfamiliar learning platform or tool, I consult with classmates about its operation methods and steps. N13: When encountering problems with online learning, I usually seek help from top-performing classmates in the class to resolve my doubts.

#### 3.2.3 Seeking social support

Some nursing undergraduates reported that they still seek social support when dealing with digital risks.

N9: Unable to find needed info online, I’ll visit school library to look for answers in books. N17: Unfamiliar tools or hard content? I’ll seek help from Tieba or Bilibili’s netizens.

### 3.3 Theme 3: learning knowledge and skills

#### 3.3.1 Nursing expertise

Given the interview proceedings, several nursing undergraduates underscored that a thorough mastery of nursing expertise forms a potent basis for tackling digital hazards.

N4: Continuous improvement of professional knowledge reserves enables avoidance of internet misinformation. N14: Essential: thorough understanding of professional courses for cross-field knowledge application & skilled nursing systems/equipment operation.

#### 3.3.2 Autonomous learning ability

Nursing undergrads interviewed concurred: Autonomous learning fosters key competence in managing digital risks.

N1: When encountering new learning platforms or tools, one should actively learn how to use them through books, online courses, and other means. N19: Online learning offers flexibility in terms of time and location, so I believe it is necessary to self-regulate and plan one’s study schedule reasonably.

#### 3.3.3 Digital technology application skills

During the interview process, a few nursing undergraduates highlighted the necessity of digital technology application skills as an indispensable basic skill for all individuals.

N7: Knowing how to use various academic tools, familiarity with each function button & location, prevents learning efficiency loss. N10: Mastery of basic computer operations ensures smooth homework completion after class. N20: Learning lit & data management software organizes info orderly.

#### 3.3.4 Digital learning skills

Some nursing undergraduates indicated that in the face of digital risks, they need to possess corresponding digital learning skills to ensure they can participate efficiently in various online learning activities.

N4: One must learn to search for materials efficiently on key websites, platforms, and databases for improved learning. N15: When faced with challenging content during online learning, knowing how to leverage AI Q&A systems for assistance is crucial.

#### 3.3.5 Digital communication skills

A minority of nursing undergraduates pointed out that cultivating digital communication skills is also one of the indispensable abilities to deal with digital risks.

N13: Master online tools for collaborative learning, actively share info with peers, enhancing learning’s efficiency & joy. N20: Proficiently communicate with teachers via email, WeChat, QQ, aiding in solving complex issues.

### 3.4 Theme 4: overcoming digital threats stress

#### 3.4.1 Psychological resilience

During interviews, nursing undergraduates largely concurred that psychological resilience is crucial for managing digital risks—upholding positivity, regulating emotions, and adapting to surroundings.

N5: Encountering novel learning software, I resist surrender & boldly tackle challenges. N8: Unable to access materials, I feel anxious yet swiftly steady, sidestepping emotional turbulence. N12: I mentally enumerate online learning’s merits, gradually embracing this study mode.

#### 3.4.2 Learning perseverance

Nursing undergraduates view learning perseverance as crucial in evading digital risks and adapting to education’s digital transformation.

N2: Essential is maintaining a thirst for learning, continually assimilating novel knowledge, and striving to refine one’s online learning abilities. N16: Encountering difficulties in utilizing a plethora of learning tools has underscored that skill mastery does not happen overnight; it necessitates a profound dedication of time towards perpetual learning endeavors.

### 3.5 Theme 5: adapt to digital environment

#### 3.5.1 Self-regulation and motivation efficacy

Nursing undergraduates noted self-regulation and motivation efficacy boosts behavioral skills, enhancing confidence in digital risk management.

N1: Without confidence in handling online learning info, one loses direction in the learning journey. N12: Lacking belief in emotion regulation, one easily falls into a whirlpool of negativity, hard to escape. N18: Recognizing digital tools’ importance in nursing sparks learning motivation.

#### 3.5.2 Online learning self-efficacy

Given the prevalence of digital risks, nursing undergraduates emphasize the significance of consistently improving their online learning self-efficacy and reinforcing their confidence in mastering and succeeding via digital resources and tools.

N5: Believing in one’s ability to manage time and resources effectively for online learning boosts efficiency. N17: Trust in one’s capacity to comprehend and master online course content aids in achieving learning goals successfully.

#### 3.5.3 Digital skills self-efficacy

Nursing undergrads noted that bolstering digital skill self-efficacy aids in resisting digital risks.

N7: Gaining confidence in proficiently using online learning platforms and software sparks motivation to learn. N20: Confident in using databases like CNKI & Wanfang notably boosts lit retrieval efficiency & enhances learning outcomes.

#### 3.5.4 Social interaction self-efficacy

During the interview process, some nursing undergraduates referenced social interaction self-efficacy, positing its beneficial role in mitigating digital risks.

N11: In online peer interactions, confident self-expression sparks problem-solving ideas. N14: Belief in barrier-free teacher communication enables active online classes participation, shifting from passive to active.

## 4 Discussion

### 4.1 Understanding digital threats

Understanding digital threats is the primary prerequisite for nursing undergraduates’ awareness of the challenges they face in the process of online learning and the application of digital resources and tools, which is the foundation for the formation of their digital resilience. This study reveals that the primary challenge encountered by these students in this process is information overload, a common sentiment expressed as the struggle to efficiently process and transform the vast expanse of information, resulting in a significant cognitive burden during decision-making processes. Importantly, the study underscores the intricate interplay between cognitive load, learning engagement, and social interaction (16, 17). Nursing undergraduates emphasize that the accrual of cognitive load has a direct and detrimental impact on their learning engagement, diminishing the depth of their online learning experiences. Concurrently, diminished learning engagement gives rise to impaired social interaction, hindering effective communication and collaboration with educators and peers. Moreover, these two factors converge to exacerbate digital technology failures and security risks, initiating a series of chain reactions that ultimately compromise the online learning effectiveness of nursing undergraduates. This underscores the paramount importance of managing information overload.

The genesis of information overload is multifaceted, intertwined with factors such as individual experience, knowledge reserves, and competency levels (18). Consequently, it is imperative for medical colleges to offer specialized training programs that systematically impart information retrieval and evaluation skills to nursing undergraduates, thereby nurturing their information and data literacy. Furthermore, these institutions should organize simulation exercises and case studies to deepen students’ understanding of the challenges posed by online learning and the utilization of digital resources and tools. By doing so, a robust foundation will be established for nursing undergraduates to effectively navigate and respond to digital risks in the future.

### 4.2 Knowing coping strategies

Knowing coping strategy is the extent to which nursing undergraduates knowing the approaches and resources available for dealing with digital risks, which is a core element in the construction of their digital resilience. A study reveals that nursing undergraduates, confronted with digital risks, typically seek assistance from teachers as their first resort. They anticipate that, under the professional tutelage and assistance of teachers, they can embark on online learning and harness digital resources and tools more efficiently. Nevertheless, the extent of teachers’ digital proficiency has a notable impact on the development of students’ digital skills (19). Consequently, it is imperative that medical colleges systematically offer professional training to teachers, encompassing the most recent educational technology tools, online teaching methodologies, and effective strategies for managing digital risks within the classroom, ensuring they remain abreast of the digital evolution in education.

Peer support, conceptualized as an exchange of emotions, sharing of information, and mutual assistance among individuals sharing similar backgrounds (20), is widely embraced by nursing undergraduates as an effective and trustworthy means. It fortifies not only the efficacy of online learning but also markedly enhances the quality of communication and interaction, corroborating previous research findings (21, 22). Social support, which refers to the assistance an individual receives from groups or social institutions, serves as a buffer against the repercussions of stressful situations and fosters adaptation (23). Recognizing their significance as a vital incubator for nursing undergraduates, medical colleges must prioritize the enhancement of campus network support services, establishing an efficient and responsive feedback system. This will enable them to provide comprehensive support and guidance to nursing undergraduates, empowering them to adeptly confront digital risks.

### 4.3 Learning knowledge and skills

The process of learning knowledge and skills for nursing undergraduates involves integrating their online learning experiences with the proficient utilization of digital resources and tools. This integration facilitates the mastery of competencies necessary to effectively address digital risks, thereby establishing a sturdy foundation for the nurturing of their digital resilience. Nursing undergraduates universally recognize that a profound foundation in professional knowledge acts as a cornerstone, enabling them to competently confront digital challenges. Concurrently, they underscore the indispensable role of self-directed learning abilities in refining learning strategies, optimizing outcomes, and serving as the primary defense mechanism against digital risks. Empirical studies have revealed a positive correlation between self-directed learning capabilities and facets such as metacognition, self-esteem, and interpersonal communication skills (24). Consequently, medical colleges must actively pursue initiatives aimed at fostering nursing undergraduates’ self-directed learning abilities by reinforcing these three integral aspects. By doing so, they empower students to engage more effectively in online learning environments.

Furthermore, nursing students perceive digital technology application ability as a fundamental prerequisite for proficient utilization of digital tools. This competency encompasses two pivotal skills: digital learning and digital communication (1). Digital learning ability emphasizes the utilization of digital platforms to access, evaluate, and manage educational resources, while digital communication ability underscores the importance of effective communication and interaction through these tools. These three elements are intricately intertwined, constituting a pivotal skill set for the digital transformation of nursing undergraduate education.

This skill system is paramount in empowering nursing students to swiftly identify pertinent learning materials, actively engage in online discussions, and ultimately enhance the efficiency and quality of online learning interactions (25). Thus, nurturing nursing undergraduates’ digital technology application ability is imperative. Studies like Ang et al. (26) have demonstrated notable outcomes by intervening in students’ abilities through their tailored RISE program, inspiring the development of specialized digital systems to strengthen nursing students’ digital technology application, digital learning, and communication skills, as well as their resilience against digital risks.

### 4.4 Overcoming digital threats stress

Overcoming digital threats stress refers to the nursing undergraduates taking appropriate regulatory measures to recover to a normal learning state after experiencing negative digital risk events, which is a key step in enhancing their digital resilience. Psychological resilience, which refers to an individual’s capacity to adapt and thrive in the face of adversity (27), is regarded by nursing undergraduates as a crucial ability to cultivate a positive mindset when confronted with digital risk stress. Current research affirms a positive correlation between psychological resilience and the perceived level of social support among nursing students (28), signifying that as their perceived social support intensifies, so does their capacity to regain a positive state amidst digital risks. This discovery underscores the pivotal role of social support in fostering psychological resilience and underscores the rationale behind considering social support as a potent strategy for nursing undergraduates to navigate digital risks in this study.

Furthermore, learning perseverance, defined as the ability to overcome uncertainties, complete courses, and maintain a commitment to learning (29, 38), is seen by nursing undergraduates as a qualification that can inspire them to proactively delve into various learning resources, enhance their professional knowledge and skills, and consequently address digital risks with greater confidence. Research reveals that the ability of online learners to sustain their learning journey is influenced by a multitude of factors, including learner characteristics, teacher attributes, course qualities, and technological platform features (29, 30). Therefore, it is highly recommended that medical colleges and universities embark on a “supply-side” reform of online education, ensuring a profound understanding of the specific needs of nursing undergraduates pertaining to course content, learning methodologies, and learning processes. Subsequently, they should strategically optimize course categories and content, teaching methods, and technological platforms, with the aim of bolstering nursing students’ inclination towards learning perseverance.

### 4.5 Adapt to digital environment

Adapting to a digital environment involves enhancing or improving nursing undergraduate students’ capacity or readiness to manage digital risks after they have progressed through the aforementioned stages of development. This process necessitates the cultivation of intrinsic confidence. The study indicates that nursing undergraduate students view self-regulation, motivation efficacy, online learning self-efficacy, digital skills self-efficacy, and social interaction self-efficacy as crucial factors in achieving successful adaptation. Self-regulation and motivational efficacy, comprising an individual’s autonomous regulation, active participation, and unwavering confidence in task completion within learning contexts (31, 32), are regarded by nursing undergraduates as the fundamental driving force that propels them to consistently employ self-regulatory strategies in their learning endeavors, particularly when confronted with digital risks and the pressures they entail.

Similarly, online learning self-efficacy, which encapsulates an individual’s subjective conviction in their ability to successfully attain objectives within online learning environments (33), is perceived by nursing undergraduates as a pivotal factor that inspires them to actively confront digital risks and enhance their online learning efficiency. Research has illuminated a strong correlation between online learning self-efficacy and the perception of teacher support (34). This study unequivocally posits that teacher support serves as the primary means for nursing undergraduates to navigate online learning challenges. It is further posited that teacher support acts as a catalyst, bolstering online learning self-efficacy and, in turn, enhancing the digital learning capabilities of nursing undergraduates. Given this understanding, there is a compelling rationale for conducting future empirical research to validate these assertions.

Additionally, digital skill self-efficacy encapsulates an individual’s confidence in their capacity to proficiently employ digital tools for the purposes of information selection, evaluation, and integration (35). Among undergraduate nursing students, it is widely believed that the activation of this belief serves to bolster their confidence and competence in utilizing digital technology, subsequently fostering the enhancement of social interaction self-efficacy. This, in consequence, translates into more assertive and confident behavior during interactions with both teachers and peers, which is congruent with previous research endeavors (36, 37). Ultimately, this chain of positive repercussions reinforces the validity of this study’s stance on the paramount importance of digital technology application skills.

## 5 Conclusion

In the digital age, where opportunities and challenges coexist, it is imperative to conduct a comprehensive assessment and nurture digital resilience among nursing undergraduates. This key measure ensures their seamless adaptation to the realm of online learning models and proficient utilization of digital resources and tools, ultimately fostering self-improvement and long-term professional development. The present study serves as a pivotal reference for establishing an evaluation system specifically tailored for assessing digital resilience among nursing students, and concurrently offers theoretical underpinnings that guide the formulation of effective training strategies during the digital transformation phase of medical colleges.

### 5.1 Limitation

To ensure the comprehensiveness and universal applicability of the data collected, it is imperative to note that the interview subjects in this study were exclusively sourced from a single higher medical college. Consequently, for future research endeavors aimed at advancing and refining the digital resilience characteristic elements among undergraduate nursing students, it is advisable to broaden the scope of participation to encompass a more diverse range of institutions.

## Data Availability

The raw data supporting the conclusions of this article will be made available by the authors, without undue reservation.

## References

[1] Ming L Jiangang C Xibin H. Research report on digital transformation of higher education teaching and learning. Beijing: Research Institute of Education, Tsinghua University (2022).

[2] Brennan PF Bakken S. Nursing needs big data and big data needs nursing. J Nurs Scholarsh. (2015) 47:477–84. doi: 10.1111/jnu.12159 26287646

[3] Pepito JA Locsin R. Can nurses remain relevant in a technologically advanced future? Int J Nurs Sci. (2019) 6:106–10. doi: 10.1016/j.ijnss.2018.09.013, PMID: 31406875 PMC6608671

[4] Heng Z Lixia C Shizheng D. A Ricoeur-inspired phenomenological-hermeneutic study of baccalaureate nursing students' online learning experience. Chin J Nurs Educ. (2022) 19:1091–5. doi: 10.3761/j.issn.1672-9234.2022.12.007

[5] Zeyu P Zi Y Faying W Yuying F. Online and offline blended learning experience of undergraduate nursing students: a meta-synthesis of qualitative studies. Chin J Nurs Educ. (2022) 19:886–893. doi: 10.3761/j.issn.1672-9234.2022.10.004

[6] Yumei S Hongyu S Hua J Hua C. The blended learning experience of nursing undergraduates in a hybrid-flexible course model. Chin J Nurs Educ. (2022) 19:869. doi: 10.3761/j.issn.1672-9234.2022.10.001

[7] Sun H Yuan C Qian Q He S Luo Q. Digital resilience among individuals in school education settings: a concept analysis based on a scoping review. Front Psych. (2022) 13:858515. doi: 10.3389/fpsyt.2022.858515, PMID: 35432032 PMC9008236

[8] Grant C Clarke C. Digital resilience: a competency framework for agile workers. Agile working and well-being in the digital age (2020). Cham: Springer International Publishing, 117–30.

[9] Zhixian Z Lihong L. Teachers’ digital resilience: connotations, framework and pathways. J Teach Educ. (2023) 6:68–74. doi: 10.13718/j.cnki.jsjy.2024.03.008

[10] Xiaoqi X Xiaowei Z Shusheng S. Break through the crisis: the digital resilience of the learning subject and its construction. E-educ Res. (2022) 43:49–55. doi: 10.13811/j.cnki.eer.2022.02.007

[11] Jianwei C Mengru X Qian F. Research on the digital resilience scale and influencing factors for university students. China Educ Technol. (2023) 6:68–74. doi: 10.3969/j.issn.1006-9860.2023.06.009

[12] Mengting J Yan Y. A review describing the methodology of qualitative studies. PLA J Nurs. (2018) 35:32–5. doi: 10.3969/j.issn.1008-9993.2018.11.008

[13] Yang L Qi L Zhang B. Concepts and evaluation of saturation in qualitative research. Adv Psychol Sci. (2022) 30:511–21. doi: 10.3724/SP.J.1042.2022.00511

[14] Huazhen L Yuanyuan J Huiling L. Research progress on the application of 3 common types of content analysis in the qualitative studies of nursing. Chin J Nurs. (2024) 59:1405–9. doi: 10.3761/j.issn.0254-1769.2024.11.018

[15] Tong A Sainsbury P Craig J. Consolidated criteria for reporting qualitative research (COREQ): a 32-item checklist for interviews and focus groups. Int J Qual Health Care. (2007) 19:349–57. doi: 10.1093/intqhc/mzm042, PMID: 17872937

[16] Shuang L Zhixia H Caoyuan C Xiaohui C. The moderating effect of cognitive load on learning engagement mechanisms in a blended learning context. Open Educ Res. (2023) 29:90–100. doi: 10.13966/j.cnki.kfjyyj.2023.04.009

[17] Qi L Juan N. A study on key factors of promoting college student engagement. J Educ Stud. (2020) 16:117–2. doi: 10.14082/j.cnki.1673-1298.2020.06.013

[18] Qiong C Yuxiang Z Shijie S Qinghua Z. Information overload in an online health information seeking context. J China Soc Sci Tech Inf. (2022) 41:424–36. doi: 10.3772/j.issn.1000-0135.2022.04.009

[19] Chuanjuan F. Building tomorrow’s digital skills—what conclusions can we draw from international comparative indicators. Journal of World Education (2017) 33:31–35.

[20] Li Z Hongxia W Jingyu G Li L. Cyberbullying victimization and depression among college students: the moderating roles of psychological capital and peer support. Journal of Psychological Science. (2024) 47:981–989. doi: 10.16719/j.cnki.1671-6981.20240427

[21] Kim-Godwin YS Turrise S Lawson S Scott M. Student perceptions of peer evaluation in an online RN-to-BSN course. Nurse Educ. (2018) 43:317–21. doi: 10.1097/NNE.0000000000000519, PMID: 29595566

[22] Brown LG Cicchino A. Asynchronous peer review feedback in an undergraduate nursing course: what students can teach each other about writing. Nurse Educ. (2022) 47:303–7. doi: 10.1097/NNE.0000000000001207, PMID: 35503114

[23] Minjiang D Youguo L. Latent class of medical freshmen’s school adjustment and the relationship among its transition, social support and mental resilience. Chin J School Health. (2024) 45:679–83. doi: 10.16835/j.cnki.1000-9817.2024158

[24] Juan D Wenkai Z Meifang W Juan L Chunping N Chunni H. Research on the Relationship between Metacognition and Self-directed Learning Ability of Undergraduate Nursing Students: A Moderated Mediation Mode. Chinese Medical Ethics. (2023) 36:1273–1280. doi: 10.12026/j.issn.1001-8565.2023.11.16

[25] Dumford AD Miller AL. Online learning in higher education: exploring advantages and disadvantages for engagement. J Comput High Educ. (2018) 30:452–65. doi: 10.1007/s12528-018-9179-z

[26] Ang WHD Shorey S Zheng ZJ Ng WHD Chen ECW Shah LBI. Resilience for undergraduate students: development and evaluation of a theory-driven, evidence-based and learner centered digital resilience skills enhancement (RISE) program. Int J Environ Res Public Health. (2022) 19:12729. doi: 10.3390/ijerph191912729, PMID: 36232028 PMC9564580

[27] Mcdermott RC Fruh SM Williams S Hauff C Graves RJ Melnyk BM. Nursing students’ resilience, depression, well-being, and academic distress: testing a moderated mediation model. J Adv Nurs. (2020) 76:3385–97. doi: 10.1111/jan.14531, PMID: 33009859 PMC7962244

[28] Yuanyuan L Changqing F. The mediating effect of school belonging between perceived social support and mental resilience in nursing undergraduates. Chin J Nurs Educ. (2024) 21:47–52. doi: 10.3761/j.issn.1672-9234.2024.01.008

[29] Lei Z Jiayu F Shuren G. A study on the impact factors of continuous learning behavior among college students in an online education environment. Heilongjiang Res High Educ. (2021) 39:141–6. doi: 10.3969/j.issn.1003-2614.2021.02.025

[30] Jing Z Huan W. Research on factors of affecting learners’ continuous learning behavior on the online education platform based on ISM. China Educ Technol. (2018) 10:123–30. doi: 10.3969/j.issn.1006-9860.2018.10.017

[31] Zhang Y. Relationships among self-esteem, self-determined motivation, self-efficacy for self-regulation and academic procrastination in health profession sunder graduates Shandong University MA thesis (2018). doi: 10.7666/d.Y3407783

[32] Teng L Zhang LJ. The influence mechanism of motivational beliefs and positive psychological traits on self-regulated learning strategies from the perspective of social cognition. J Xi’an Int Stud Univ. (2024) 32:38–44. doi: 10.16362/j.cnki.cn61-1457/h.2024.03.003

[33] Ya S Yongbing L Tingting S Jianglin Z Jingxin Z Ran Y. Correlation and influencing factors between online learning engagement and online learning self-efficacy of medical students. J Nurs Adm. (2021) 21:334–9. doi: 10.3969/j.issn.1671-315x.2021.05.007

[34] Xie Y. Research on the influence of undergraduate perceive support from teacher on undergraduate online learning self-efficacy Hunan University (2023). doi: 10.27135/d.cnki.ghudu.2023.002041

[35] Wang K. ICT self-efficacy and learning adaptation: moderated mediation model Central China Normal University (2021). doi: 10.27159/d.cnki.ghzsu.2021.000705

[36] Liu M Zuo J Tao Y Zhao L Wu S Feng L. Influencing factors of learning sustained attention for nursing students in online settings: a structural equation model. Nurse Educ Today. (2022) 111:105248. doi: 10.1016/j.nedt.2021.105248, PMID: 35247674

[37] Demirelli EG Karaçay P. Factors associated with nursing students' online learning self-efficacy: a descriptive cross-sectional study. Nurse Educ Today. (2024) 132:106029. doi: 10.1016/j.nedt.2023.106029, PMID: 37976885

[38] Yan L Xiu G Xiaoqing G. Shall We Really Know about Perseverance? A Malleable Model from the Viewing of Teachers’ Based on Grounded Theory. Global Education. (2022) 51:39–58.

